# The Effect of Threat on Novelty Evoked Amygdala Responses

**DOI:** 10.1371/journal.pone.0063220

**Published:** 2013-05-03

**Authors:** Nicholas L. Balderston, Doug H. Schultz, Fred J. Helmstetter

**Affiliations:** 1 Department of Psychology, University of Wisconsin-Milwaukee, Milwaukee, Wisconsin, United States of America; 2 Department of Neurology, Medical College of Wisconsin, Milwaukee, Wisconsin, United States of America; George Mason University/Krasnow Institute for Advanced Study, United States of America

## Abstract

A number of recent papers have suggested that the amygdala plays a role in the brain’s novelty detection circuit. In a recent study, we showed that this role may be specific to certain classes of biologically-relevant stimuli, such as human faces. The purpose of the present experiment was to determine whether other biologically-relevant stimuli also evoke novelty specific amygdala responses. To test this idea, we presented novel and repeated images of snakes and flowers while measuring BOLD. Surprisingly, we found that novel images of snakes and flowers evoke more amygdala activity than repeated images of snakes and flowers. Our results further confirm the robustness of the novelty evoked amygdala responses, even when compared with effects more traditionally associated with the amygdala. In addition, our results suggest that threatening stimuli may prime the amygdala to respond to other types of stimuli as well.

## Introduction

Exposure to novel stimuli evokes activity in a network of structures important for learning and memory [1]–[11]. Although early investigations tended to focus on the hippocampus as the brain’s primary novelty detector [1]–[6], recent studies have shown that the amygdala also responds to novelty under certain circumstances [7]–[11]. In a previous functional magnetic resonance imaging (fMRI) study we explored the circumstances that were important for amygdala novelty responses by comparing novel and repeated images, and manipulating the content of the images [12]. We showed that both emotional and neutral images evoke more amygdala activity when novel than when repeated, suggesting that emotional content was not necessary for novelty specific amygdala responses. In a second experiment, we showed that neutral images of humans, but not scenes, evoke more amygdala activity when novel than when repeated, suggesting that the presence of a human representation was necessary for novelty specific amygdala responses.

Unlike the amygdala, the hippocampus responded more to all novel stimuli in our experiments, suggesting that it plays a general role in novelty detection. The results from that study suggest that the amygdala and hippocampus play different roles in novelty detection. While the hippocampus seems to play a general role in novelty detection, the amygdala seems to play a stimulus-specific role in novelty detection. This stimulus specificity may be related to our need to use human faces as evidence for threats in the environment [13].

Having previously shown that only certain types of stimuli evoke amygdala novelty responses, we wanted to determine the nature of this stimulus specificity. We hypothesized that these responses are not limited to faces, but are specific to a larger class of biologically-relevant stimuli that have predicted significant outcomes in the evolutionary and personal history of the subject [14], and that the function of these responses is to evaluate novel biologically-relevant stimuli for evidence of threat in the environment. To test this hypothesis, we presented novel and repeated images of biologically-relevant and control stimuli while measuring brain activity with fMRI. We chose snakes as a biologically-relevant class of stimuli because these animals evoke fear responses in primates that are abolished following lesions of the amygdala [15]–[17]. We chose flowers as our control stimuli because share many contextual elements with the snake images, and they have been previously used as control stimuli in similar behavioral experiments [18]–[20]. If the biological-relevance hypothesis is correct, snakes but not flowers should evoke a novelty response in the amygdala.

## Materials and Methods

### Participants

Twenty-three neurologically healthy undergraduate students (Age: M = 21.72, SD = 3.56) at the University of Wisconsin-Milwaukee participated in this experiment and received $20 for participation, as well as extra credit in their psychology classes and a picture of their brain. Twelve were female. One participant withdrew from the experiment during scanning, one was excluded because of excessive head motion, and three were excluded because of equipment malfunction. All participants gave informed consent, and the protocol was approved by the Institutional Review Boards for human subject research at the University of Wisconsin-Milwaukee and the Medical College of Wisconsin.

### Procedure

**Figure 1:**
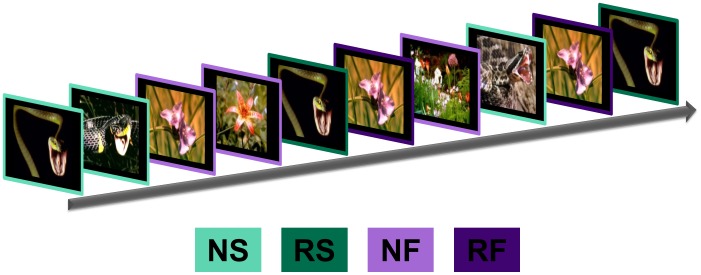
We presented novel and repeated images of snakes and flowers while measuring BOLD activity. (**a**) We presented images sequentially for 8 seconds each in an event related design. All participants saw 5 presentations of novel snake (NS) and 5 presentations of novel flower (NF) images indicated by the light green and light purple outlines, respectively. In addition all saw 5 repetitions of one snake (RS) and one flower (RF) image, shown in dark green and dark purple respectively.

During the experiment, we presented a series of 20 eight-second novel and repeated images (See Figure 1), using the same procedures as in a previous study [12]. The novel conditions included five presentations of different images, where each image was presented only once. The repeated conditions included five presentations of a single image, which was counterbalanced across subjects. For the repeated conditions we included only trials where the image had been repeated. Therefore, the initial presentation of these stimuli was counted in the respective novel categories. We also manipulated the content of the stimuli by presenting images of snakes (novel = NS; repeated = RS) and flowers (novel = NF; repeated = RF) from the International Affective Picture System (IAPS) database [21]. See Figure 1 for design summary and Table 1 for affective ratings.

**Table 1:** IAPS Normative Ratings.

Slide Number	Valence	Arousal
*Flower*		
5000	7.08	2.67
5010	7.14	3.00
5020	6.32	2.63
5030	6.51	2.74
5200	7.36	3.20
*Snake*		
1026	4.09	5.61
1050	3.46	6.87
1052	3.50	6.52
1113	3.81	6.06
1114	4.03	6.33

We presented the stimuli using the software package Presentation (Neurobehavioral Systems, Inc., Albany, CA), on a Dell laptop (model: Inspiron 9300, Dell Inc., Red Rock, TX). Participants viewed the stimuli via a back projection system with prism glasses mounted to the head coil. Each picture was presented centrally, and presentations were separated by a 20 second average variable intertrial interval (ITI; ±4 sec).

Prior to the experiment, we situated the participant comfortably in the scanner, secured their head with cushions, and attached the physiological monitoring equipment. As part of a separate experiment, we exposed participants to several presentations of an aversive electrical stimulation. During the experiment we measured their expectancy of receiving the electrical stimulus; however, the stimulus was never presented during the experimental phase. We also measured skin conductance throughout the experiment. After the experiment, participants were removed from the scanner and asked to complete a post experimental questionnaire.

### MRI

We conducted whole brain imaging using a 3 Tesla short bore GE Signa Excite MRI system. Functional images were acquired using a T2* weighted gradient-echo, echoplanar pulse sequence. Contiguous four millimeter sagittal slices (TR = 2 sec; TE = 25 ms; field of view = 24 cm; flip angle = 90°) were collected during the experiment. We collected 290 whole brain images. In addition to the functional images, we also collected high resolution spoiled gradient recalled (SPGR) acquisition images to serve as a three-dimensional anatomical map for the functional images.

### MRI Segmentation

We performed the subcortical segmentation using Freesurfer software package, which is freely available online and has been described previously [22], [23]. Freesurfer generated volumes were then realigned to native space using The Analysis of Functional NeuroImages software package (AFNI). These realigned volumes were then manually inspected to ensure that they conformed to previously described standards [24].

### Functional Imaging Data Acquisition

Functional imaging data were reconstructed and processed using AFNI [25]. fMRI data were passed through motion correction and edge detection algorithms, then registered to the fifth image in the timeseries. Raw fMRI data were manually inspected for large head movements. Images that contained discrete head movements were censored, and participants showing excessive movement (greater than 2 mm displacement or more than 5 instances of discrete head movements) were excluded from further analyses. Head motion and dial movement regressors were included in the analysis as regressors of no interest. Timeseries data were deconvolved with stimulus canonicals using a least squares procedure, to yield average impulse response functions (IRFs).

### Functional Imaging Data Analysis

For whole brain analyses SPGR images were manually warped into Talairach space using anatomical markers. Images one through four of the IRFs were used to calculate percent area under the curve (%AUC). These images were chosen because they correspond to the stimulus presentation plus a two second delay (image zero) to account for the delayed onset of the hemodynamic response. The %AUC maps were then registered to Talairach space and resampled to 1 mm isotropic voxels using linear interpolation. Images were then blurred using a 4 mm full-width at half-maximum Gaussian kernel. The resulting maps were used in the group level analyses. We used the AFNI program AlphaSim to determine an appropriate cluster threshold to correct for multiple comparisons across the voxels in the whole brain volume (*p* = 0.005; rmm = 2; xyz = 1; Volume = 228 µL; corrected *α* = 0.05).

For the ROI analyses, image three from the IRF was registered to the unwarped SPGR data on a subject-by-subject basis and resampled to 1 mm isotropic voxels using linear interpolation. Image three was chosen for the ROI analysis because in our previous novelty studies this image corresponded to the peak of the hemodynamic response function in the amygdala. The images used for the ROI analyses were not warped or blurred, in order to forego the distortion caused by these procedures. Because voxelwise data were not used in the group-level analyses, these steps were unnecessary. We chose an alpha level of 0.05 for all analyses.

### Skin Conductance Responses

As in previous experiments [12], [26]–[33], we recorded skin conductance level (SCL) via two surface cup electrodes (silver/silver chloride, 8 mm diameter, Biopac model EL258-RT, Goleta, CA) filled with electrolyte gel (Signa Gel, Parker laboratories Fairfield, NJ) attached to the bottom of the participants left foot approximately 2 cm apart, and sampled at 200 Hz throughout the experiment. We sampled SCL during the 8 second stimulus period and the preceding two second baseline period. Raw values for each trial were normalized to that trial’s average baseline SCL and expressed as a percent change from that baseline value. We used the maximum value within the stimulus period to represent the response magnitude for each trial. Statistical analyses were performed on that value. We chose an alpha level of 0.05 for all analyses.

### Electrical Stimulation

Prior to the experiment, participants were given presentations of an electrical stimulation. Electrical stimulation was administered via an AC (60 Hz) source (Contact Precision Instruments, Model SHK1, Boston, MA) through two surface cup electrodes (silver/silver chloride, 8 mm diameter, Biopac model EL258-RT, Goleta, CA) filled with electrolyte gel (Signa Gel, Parker laboratories Fairfield, NJ). The electrodes were placed on the skin over the subject’s right tibial nerve over the right medial malleolus. Participants were given several half-second presentations of the shock. They rated the shock on a scale from zero (no sensation) to ten (painful but tolerable). Intensity was increased gradually in mA until participants rated the sensation as a ten.

### Shock Expectancy

Participants continuously rated their expectancy of receiving the electrical stimulation during the experiment. Even though the participants never received the stimulation during this session, we used this procedure for two reasons. First, we wanted to ensure that the subjects were attending to the stimuli. Second, the purpose of this study was to replicate our previous novelty experiments using a different class of stimuli. Therefore, we wanted to keep the methodology as similar as possible. During the session, participants controlled a visual analog scale on the computer screen using a dial. The analog scale was anchored with 0 and 100. Participants were instructed to move the cursor to 0 if they were absolutely sure that they would not receive an electrical stimulation, to move the cursor to 100 if they were absolutely sure that they would receive a stimulation, and to keep the cursor near 50 if they felt like there was an equal probability of receiving or not receiving a stimulation. Responses were recorded throughout the experiment and sampled at 40 Hz. An alpha level of 0.05 was used for all analyses.

### Post-experimental Questionnaire

Following the experiment, subjects were asked to rate the arousal and valence of the images [21]. For each picture, subjects were asked two questions about how the picture made them feel. They responded to each question using a nine-point likert type scale anchored with appropriate descriptors (arousal: excited-calm; valence happy-unhappy). Pictures were presented in a random order on a laptop computer in a quiet room near the scanner. Subjects were given as much time as needed to complete the questionnaire.

## Results

### Novel Snakes and Flowers Evoke Responses in Human Amygdala

**Figure 2:**
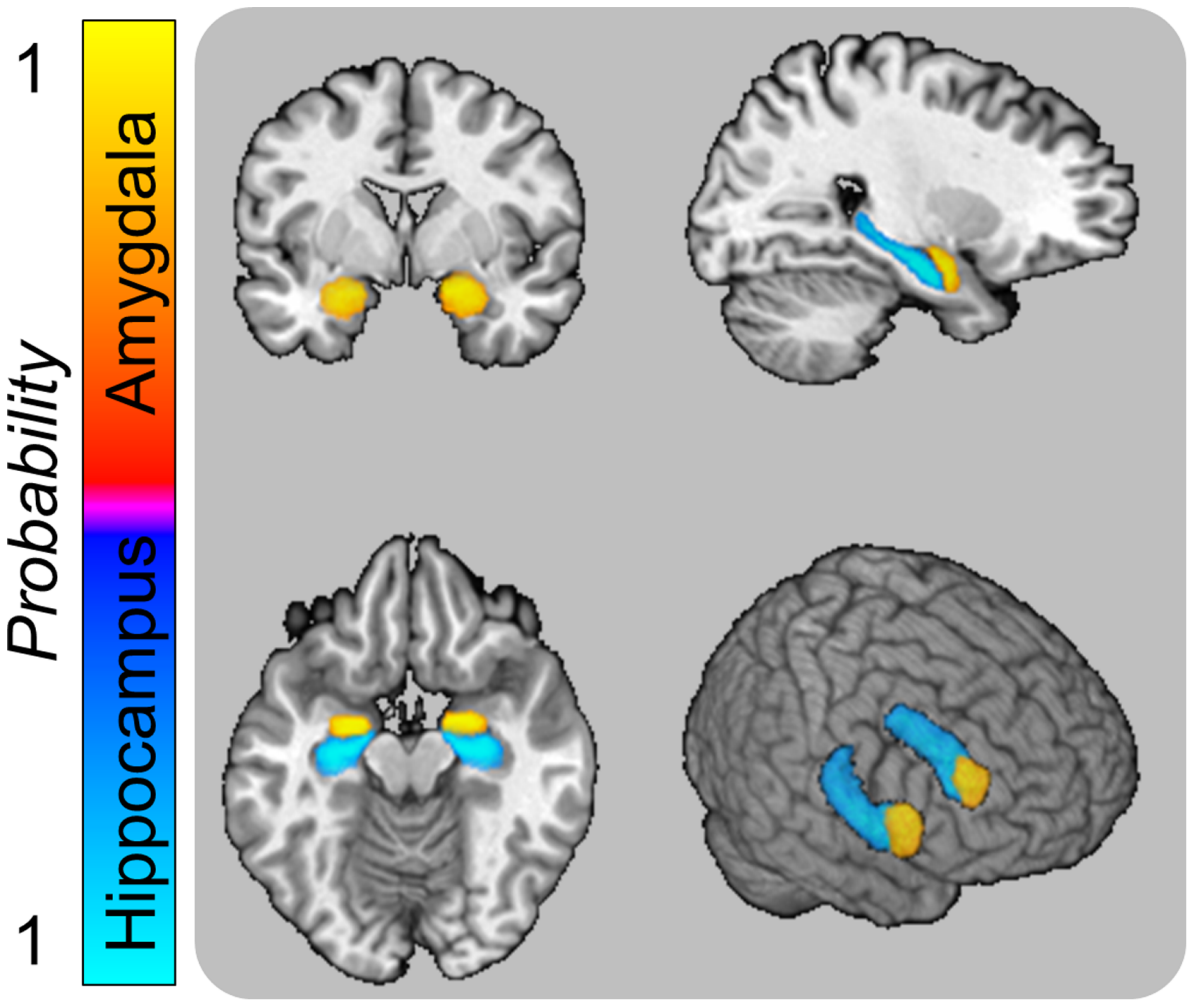
We sampled BOLD activity in the amygdala and hippocampus using anatomical regions of interest. We defined the amygdala and hippocampus for each individual using Freesurfer, and sampled BOLD in native space activity using these regions of interest. The images in this figure show the axial, sagittal, coronal, and rendered view of the amygdala and hippocampus in Talairach space, collapsed across subjects.

Because these hypotheses stem from our previously published work, we used the same procedures to present the stimuli and analyze the imaging data [12]. Briefly, we presented novel and repeated images of snakes and flowers, while measuring BOLD activity. We anatomically identified the amygdala and hippocampus using each subject’s T1 weighted volume (See Figure 2 for probability map in Talairach space). We sampled the BOLD activity in these structures during the last 2 seconds of the stimulus period. We performed a 2 (Novel vs. Repeated) × 2 (Snake vs. Flower) × 2 (Left vs. Right) repeated measures ANOVA on those values.

**Figure 3:**
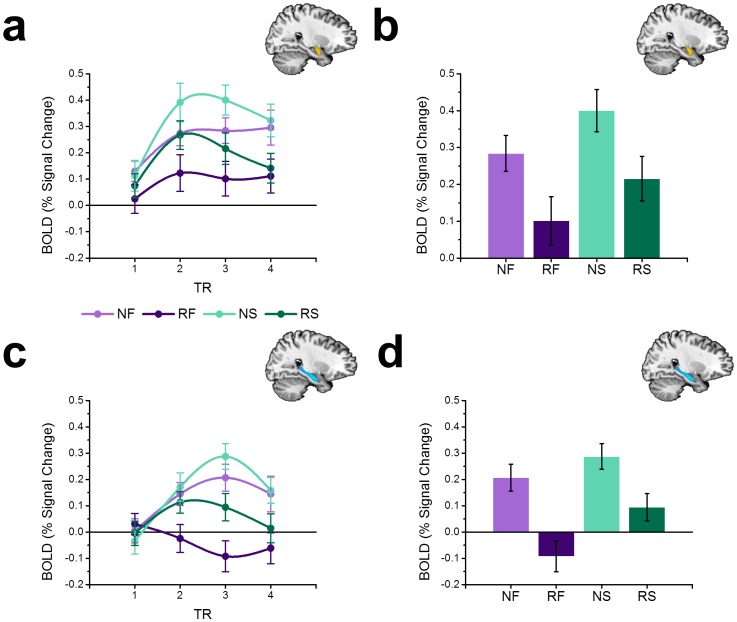
Novel snakes and novel flowers drive BOLD activity in the amygdala and hippocampus. (**a,c**) Line graphs represent BOLD timecourse in the amygdala (**a**) and hippocampus (**c**). (**b,d**) Bar graphs represent the percent signal change in the amygdala (**b**) and hippocampus (**d**) during the last two seconds of the stimulus period. All data points represent mean±SEM. (NS = novel snake, RS = repeated snake, NF = novel flower, RF = repeated flower).

Contrary to our hypothesis we did not observe a novelty × stimulus type interaction. Instead we found that snakes and flowers evoked more amygdala activity when novel than when repeated (*F*(1,17) = 9.155; *p* = 0.008; See Figure 3a,b). Although we chose to use an anatomical ROI approach to remain consistent with our previous study, results from the whole brain analysis also show significant clusters of activation within the amygdala. Snakes and flowers also evoked more hippocampal activity when novel than when repeated (*F*(1,17) = 15.690; *p* = 0.001; See Figure 3c,d), consistent with the hippocampal novelty detection hypothesis. In contrast, the effect of threat on amygdala and hippocampus responses was only a trend. Snakes evoked only marginally more amygdala (*F*(1,17) = 3.393; *p* = 0.083) and hippocampal (*F*(1,17) = 3.829; *p* = 0.067) activity than flowers. In the hippocampus, this effect seemed to be larger in the left hemisphere (*F*(1,17) = 4.822; *p* = 0.042), but there were no other significant effects (*p*s <0.1).

### Snakes Evoke More Lateral Occipital Cortex and Fusiform Gyrus Activity than Flowers

**Figure 4:**
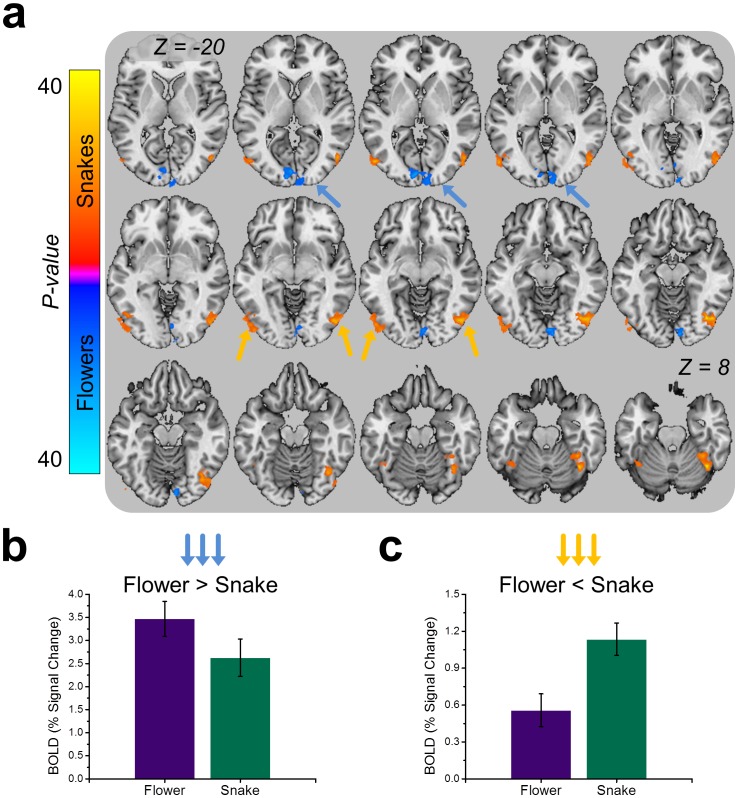
Images of snakes and flowers evoke distinct patterns of activity in visual cortical areas. (**a**) Series of axial sections displaying the results from the whole brain Snake>Flower comparison. Colors indicate size and direction of *F*-statistic depicted on brain slice, and correspond to the colors on the scale to the left. (**b**) Flowers evoke more activity than snakes in lower-level visual processing areas. (**c**) Snakes evoke more activity than flowers in higher-level visual processing areas. Bar graphs represent the percent signal change in the structures marked by the colored arrows.

In addition to sampling BOLD activity in the amygdala and hippocampus, we also performed a mixed-effects ANOVA across voxels in the entire brain (See Table 2). We used novelty and stimulus type as fixed factors, and subject as a random factor. Interestingly, flowers and snakes tended to evoke different patterns of visual cortical activity (See Figure 4). Flowers evoked more activity in early visual processing areas like the calcarine sulcus. In contrast, snakes evoked more activity in later visual processing areas like the lateral occipital cortex and the fusiform gyrus.

**Table 2:** Stimulus Type × Novelty ANOVA Effects.

Structure	Coordinates			*F*	Volume	Direction
	RL	AP	IS		(mm3)	
*Stimulus Type*						
Right Lateral Occipital Gyrus/Fusiform Gyrus	−42	63	−10.80	15.33	3757	S>F
Left Lateral Occipital Gyrus	48	72	−3.20	13.08	1515	S>F
Bilateral Calcarine Sulcus	−1	89	4.20	12.78	1168	F>S
Right Lingual Gyrus	−5	83	−7.60	13.30	592	F>S
Left Fusiform Gyrus	38	52	−19.30	11.73	252	S>F
*Novelty*						
Left Inferior Frontal Gyrus/Temporal Pole	35.8	−23.5	−16.1	13.35	1554	N>R
Right Lingual Gyrus	−18.4	77.6	−15.9	13.86	982	N>R
Left Superior Frontal Gyrus	9.1	−25.1	60.6	12.43	961	N>R
Right amygdala/Substantia Innominata	−14.1	0.2	−10.6	14.97	829	N>R
Right Lateral Occipital Gyrus	−37.9	72.4	−9.8	11.97	564	N>R
Left Insula	44.3	−17.2	−2.8	13.50	527	N>R
Left Fusiform Gyrus	35	36.5	−21.6	13.92	507	N>R
Right Middle Occipital Gyrus	−16.7	91.5	16.5	12.23	411	N>R
Left Amygdala	18.9	1.7	−11.8	16.10	349	N>R
Right Inferior Temporal Gyrus	−48.1	68.8	0.8	11.92	347	N>R
Right Superior Parietal Lobule	−26	61.5	56.5	13.62	305	N>R
Left Superior Frontal Gyrus	13.5	−54.4	31.8	12.18	290	N>R
Right Lingual Gyrus	−19.5	85.4	0.8	12.61	255	N>R
Left Posterior Cingulate	3.6	46.2	14.9	12.31	229	N>R

### Snakes are More Arousing and Threatening than Flowers

**Figure 5:**
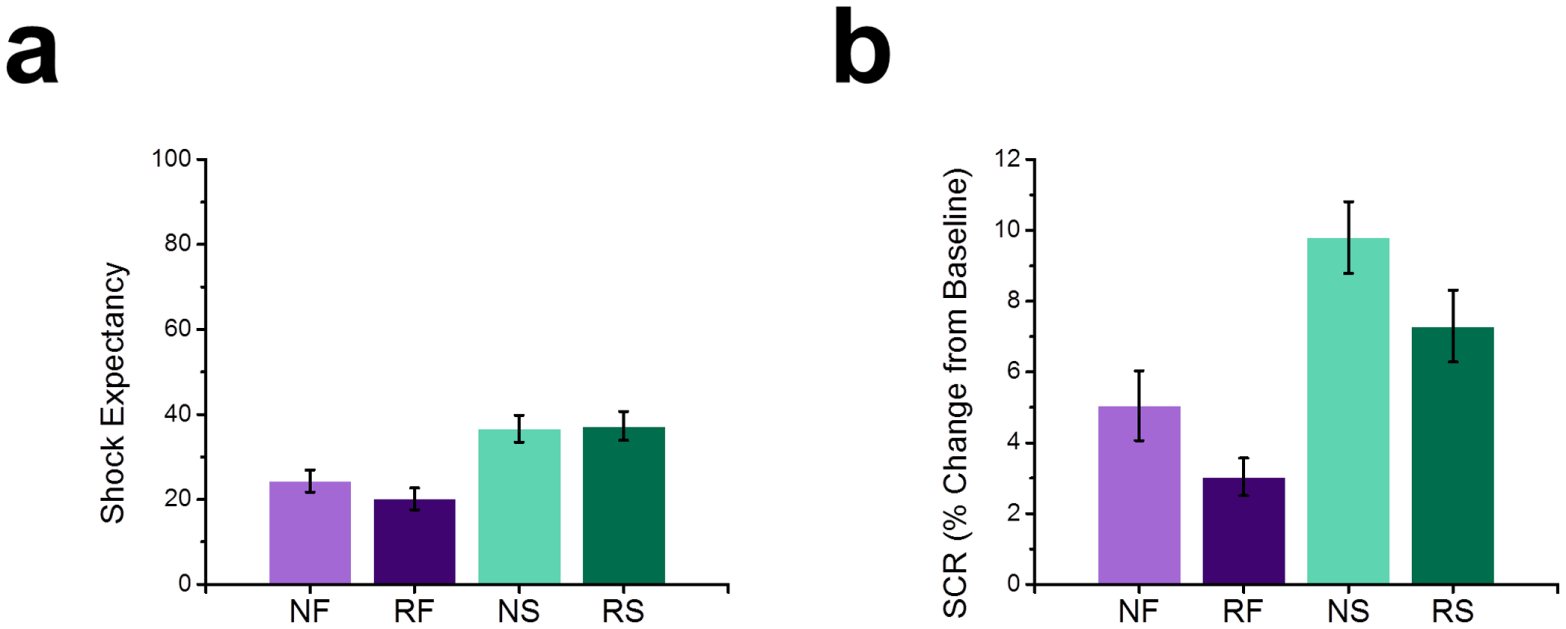
Snakes were perceived as more threatening, and evoked larger SCRs than flowers. (**a**) Participants rated snakes as more likely to predict an electrical stimulation than flowers. (**b**) Participants showed larger SCRs to snake images than flower images. Participants also showed larger SCRs to novel images than to repeated images. (SCR = skin conductance response, NS = novel snake, RS = repeated snake, NF = novel flower, RF = repeated flower).

Although amygdala responses seemed to be most sensitive to novelty, behavioral responses tended to differ based on stimulus type. Snakes were more rated as more arousing (*M* (*SEM*); Snake = 6.56 (0.245); Flower = 4.084 (0.359); *t*(17) = 5.153, *p*<0.0005), and negative (*M* (*SEM*); Snake = 6.902 (0.376); Flower = 3.700 (0.314); *t*(17) = 5.195, *p* = 0. 0005) than flowers. Likewise, subjects were more likely to expect an aversive outcome while viewing a snake than while viewing a flower (*F*(1,17) = 5.195; *p* = 0.036; See Figure 5a), and Across trials individuals learned to associate the picture with the absence of shock (*F*(1,4) = 2.717; *p* = 0.037). In addition to explicit responding, snakes also evoked larger SCRs than flowers as well (*F*(1,17) = 7.866; *p* = 0.012; See Figure 5b), even though there was a trend for SCRs to decrease in magnitude across trials (*F*(4.17) = 2.148; *p* = 0.084). Additionally, novel stimuli evoked larger SCRs than repeated stimuli (*F*(1,17) = 5.737; *p* = 0.028).

## Discussion

### Both Snakes and Flowers Drive the Novelty Effect in the Amygdala

In our original experiment we found that the amygdala preferentially responded to novel stimuli, but in a stimulus specific manner. We showed that the amygdala responded more to novel than to repeated faces, but similarly to novel and repeated scenes [12]. Based on this finding we hypothesized that the amygdala initially evaluates a stimulus for evidence of threat in the environment and that this threat evaluation occurs exclusively for stimulus types that have previously signaled threats in the personal and evolutionary history of the individual. Accordingly, for this experiment we predicted that novel snakes would evoke more amygdala activity than repeated snakes, but that novel and repeated flowers would evoke similar magnitude amygdala responses. Instead we found that both snakes and flowers evoke more amygdala activity when novel than when repeated, and this effect was robust for both the whole brain and the ROI analyses. Additionally, the effect of threat (snake>flower) on amygdala responses was only a trend. These results are consistent with our previous novelty study where we manipulated emotion, and also found only a marginal effect. Given that the amygdala has been repeatedly implicated in emotion and threat processing, it is interesting to note that the novelty effects we observe are so much more robust by comparison. Although these results were initially surprising, we believe that the amygdala novelty response represents the information processing needed to evaluate a given stimulus for evidence of threat in the environment. However, rather than being dependent exclusively on the content of the stimulus, the current results suggest that the likelihood of an amygdala novelty response is also dependent upon the probability that the individual may encounter a threat (e.g. snake).

There is a long history of research into predatory imminence [34]–[36], suggesting that animals adapt their fear response based their proximity to a given threat. The presence of a snake represents an immediate threat to the safety of the individual [37]. Primates may have an innate fear of snakes, which is abolished in non-human primates with amygdala lesions [15]–[17]. In addition, in other experiments snakes capture attention in visual displays [38], interfere with goal directed behavior [39], and lead to rapid conditioning that is more resistant to extinction [40], [41]. In the current experiment, snakes evoked larger SCRs than the flowers, were rated as more arousing and negative, and were also reported to be more likely to predict an aversive outcome, despite the fact that no programmed experimental contingency was in place. Taken together, these findings suggest that the snakes in our experiment may have represented a perceived threat, and the amygdala novelty response evoked by the flowers in our experiment may be a function of the presence of this perceived threat.

More generally, the results from our current experiment combined with the results from our prior work suggest that amygdala evaluates novel stimuli based on the combined probability that the environment or the stimulus itself could signal a potentially threatening situation. When danger is imminent, the amygdala evaluates all stimuli for evidence of potential threats. However when danger is not imminent, the amygdala only evaluates novel stimuli that have previously signaled threat in the personal and evolutionary history of the individual. This theory raises at least two testable hypotheses. First, it should be possible to show the presence and absence of novelty responses evoked by the same stimulus category under conditions of threat and no threat, respectively. Second, it should be possible to show the presence and absence of novelty responses evoked by the same stimulus category in individuals for whom that stimulus category has and has not previously signaled danger. For instance, patients with post-traumatic stress disorder (PTSD) may show amygdala novelty responses to stimuli related to their trauma, while individuals without PTSD might not.

Although the predatory imminence theory incorporates amygdala novelty responses into a previously established framework of defensive behaviors, the specific hypotheses generated by this theory have yet to be fully tested, and there are several other potential explanations for our current results. First, it is possible that valence or possibly rarity, and not biological-relevance may be important for novelty specific amygdala responses [7], [11]. Blackford and colleagues show that both novel common and uncommon stimuli evoke more amygdala and hippocampal activity than familiar stimuli, and that novel uncommon stimuli evoke more amygdala activity than novel common stimuli. Similarly, Weierich and colleagues show that novel positive and negative IAPS pictures evoke more amygdala activity than repeated stimuli. Thus it is possible that a positive or negative valence is sufficient to evoke an amygdala novelty response. In fact, the normative ratings of the flowers and scenes from the previous study were slightly different. The flowers were rated as slightly more positive and slightly more arousing than the scenes we used previously. Even though valence may be sufficient to drive novelty responses in the amygdala, it does not seem to be necessary, because the neutral faces that we used in our previous study evoked a novelty response, but neutral scenes with a similar valence did not.

In the current study we show that flowers evoke an amygdala novelty response, but in our previous study neutral scenes did not. Given that the scenes used in the previous study were mostly artificial situations with few elements seen in nature, it is also possible that the amygdala might respond only to natural scenes.

Another possible explanation is that the novelty effect we observed for flowers in the amygdala is due to the fact that the flowers represent a foreground object in the images, whereas the scenes from the previous study generally lacked foreground objects. Although there is little direct evidence that the amygdala is specifically sensitive to objects, while holding other factors constant, most of the studies showing amygdala activation to visual stimuli use discrete stimuli like faces or small animals [42]–[51]. Interestingly, one study directly compared amygdala activation to emotional faces and scenes and found that both stimulus classes evoked amygdala responses, but that faces evoke larger responses than scenes [52]. In the future, we would like to distinguish between these possibilities.

The current study is an extension of our previous novelty study [12], which in turn was adapted from our standard fear conditioning protocol. As a result, the current study, as well as our previously published study, is limited by the relatively small number of trials compared to other novelty studies [7], [11]. As a result this study is not optimally designed to detect differences in novelty responding over the course of experimental session. Given that amygdala responses have been shown to habituate over the course of an experimental run [53], future studies should be designed to investigate the possibility that amygdala novelty responses share this characteristic. In addition to the limited number of trials, our study is also limited by the presence of an aversive stimulus prior to the experiment. Although it is difficult to tell what influence this aversive stimulus has on amygdala novelty responses, previous work has shown that the presence of the aversive stimulus is not necessary for amygdala novelty responses [7], [11]. Additionally, in our previous study we found that neutral scenes did not evoke a novelty response, suggesting that the presence of an aversive stimulus is not sufficient to drive amygdala novelty responses.

### The Hippocampus Plays a General Role in Novelty Detection

Consistent with our hypothesis, the hippocampus responded maximally to novel snakes and flowers. This is the third experiment where we have seen a general novelty effect for the hippocampus. These results further support the hypothesis that the hippocampus is sensitive to novelty, independent of the perceptual characteristics of the novel stimulus [54]. Furthermore, they are consistent with several previous studies that used a wide variety of stimuli, each showing novelty effects in the hippocampus [1]–[6]. In addition to functional neuroimaging studies, there are other converging lines of evidence suggesting that the hippocampus acts as a novelty detector. For instance, single units in the hippocampus respond to novel stimuli [55] and hippocampal lesions reduce the magnitude of ERPs evoked by novel words [2] and sounds [56]. In addition to stimulus novelty, the hippocampus also seems to be sensitive to contextual novelty. In our first experiment, the hippocampal novelty response diminished with repeated trials [12]. Strange and Dolan [57] and Yamaguchi et al. [6] have also showed that hippocampal responses to novel stimuli decrease across trials.

Hippocampal function is necessary to encode new episodic memories [58], [59], and hippocampal activity during memory encoding is often correlated with memory strength [60], [61]. Novelty has been previously hypothesized to play an important role in memory encoding. The fact that the hippocampal novelty response seems to be a general phenomenon is consistent with a role in episodic memory encoding. However, a recent study from Poppenk and colleagues [62] challenges this hypothesis. In this study, the researchers presented novel and familiar scenes to subjects while recording fMRI. They then tested subjects’ memory for the scenes outside the scanner. Interestingly, they found that activity in the anterior hippocampus correlated with memory for the novel scenes, while activity in the posterior hippocampus correlated with memory for the familiarized scenes. However, contrary to the novelty-encoding hypothesis, subjects actually showed better source memory for the repeated scenes. Although interesting, these findings do not completely rule out the novelty-encoding hypothesis because the familiarized scenes were actually novel at one point during the experiment. Given that every stimulus that an individual encounters is at some point novel, it is difficult to actually test the novelty-encoding hypothesis. However, if hippocampal novelty responses actually do contribute to memory encoding, encoding should be best served when those responses are the largest. Because hippocampal novelty responses tend to diminish across trials in a given experiment [6], [12], [57], it is possible to test the novelty-encoding hypothesis by comparing memory for novel stimuli presented during early versus late trials. According to the novelty-encoding hypothesis, early trials (where the hippocampal novelty response is large) should lead to better memory than later trials (where the hippocampal novelty response is smaller). Poppenk et al. [62] found that subsequent memory for novel and repeated scenes was correlated with different hippocampal regions. Although we didn’t test for subsequent memory in our experiment, we did attempt to determine whether there were differences in novelty responding in the anterior and posterior hippocampus. We were unable to detect any differences across the different hippocampal regions.

### Snakes and Flowers each Evoke Distinct Patterns of Visual Cortical Activity

In addition to the region of interest analysis, we also performed a voxelwise ANOVA across the entire brain. We found that images of flowers activated early visual processing areas, like V1 located in the calcarine sulcus. One of the limitations of this study is that we selected our stimuli based on psychological characteristics, rather than perceptual characteristics. As a result, the differences in response magnitude evoked by the flowers in V1 may have been driven by differences in the perceptual qualities of the stimuli across categories. Although we did not quantitatively assess these differences, the flower images tended to be more complex than the snake images.

Remarkably, even though flowers evoked more activity in early visual processing areas, images of snakes still led to more activation in areas later in the ventral visual processing stream, like the lateral occipital cortex and the fusiform gyrus. These data replicate several previously published studies and suggest that threatening stimuli are processed more thoroughly by higher-level sensory cortical areas [44], [48], [63]–[65], which may be driven by reciprocal connections with the amygdala [48], [63], [66]. For instance, Glascher and colleagues [64] showed that emotional stimuli evoke larger responses than neutral stimuli in the lateral occipital cortex. Spider phobic patients show larger fusiform gyrus responses than non-phobic individuals when anticipating spider pictures [67]. Threat-related geometric shapes (downward facing triangles) evoke more fusiform gyrus and amygdala activity than non-threatening shapes (upward facing triangles), and also lead to greater functional connectivity in these areas [48]. Finally, in a recent study Sabatinelli and colleagues [63] showed that amygdala and the inferotemporal cortex differentiate between arousing and non-arousing images more rapidly than the middle occipital gyrus, which comes earlier in the visual processing stream.

Our study adds to these findings because we show that not all amygdala effects lead to increases in activity in these visual processing areas. Our results show a robust main effect for novelty and only a marginal effect for threat in the amygdala. However, we do not observe a robust effect for novelty in the lateral occipital cortex or the fusiform gyrus. Differences in activity in these areas are driven by the category of the stimulus. Thus if these visual cortical effects are driven by reciprocal connections with the amygdala, as some have hypothesized [68], then there must be distinct circuits in the amygdala sensitive to these different experimental manipulations.

### Conclusions

Here we show that novel images of snakes and flowers evoke more amygdala and hippocampus activity than repeated images of snakes and flowers. We believe that amygdala novelty responses represent an initial evaluation of the stimulus, for evidence of threat in the environment. These results combined with the results from our previous experiment suggest that this need to evaluate novel stimuli for threat signals is influenced both by the content of the stimulus, and by the context in which the stimulus is presented. Under safe conditions, the amygdala evaluates only “biologically-relevant” novel stimuli, while in unsafe conditions, the amygdala evaluates all novel stimuli.

We also show that novel images of snakes evoke more activity in the lateral occipital cortex and fusiform gyrus than images of flowers. Given that threat, not novelty, led to robust effects in high-level visual cortical regions, these results also suggest that there may be distinct neural circuits in the amygdala sensitive to these two stimulus attributes. Additionally, future research using high resolution fMRI should be conducted to determine whether novelty and threat are processed by spatially distinct neural circuits in the amygdala.
